# Synchronized development of thymic eosinophils and thymocytes

**DOI:** 10.1093/intimm/dxae037

**Published:** 2024-06-25

**Authors:** Ayami Ota, Takahiro Iguchi, Sachiko Nitta, Ryunosuke Muro, Nanami Mino, Masayuki Tsukasaki, Josef M Penninger, Takeshi Nitta, Hiroshi Takayanagi

**Affiliations:** Department of Immunology, Graduate School of Medicine and Faculty of Medicine, The University of Tokyo, Tokyo 113-0033, Japan; Department of Immunology, Graduate School of Medicine and Faculty of Medicine, The University of Tokyo, Tokyo 113-0033, Japan; Department of Immunology, Graduate School of Medicine and Faculty of Medicine, The University of Tokyo, Tokyo 113-0033, Japan; Department of Immunology, Graduate School of Medicine and Faculty of Medicine, The University of Tokyo, Tokyo 113-0033, Japan; Department of Immunology, Graduate School of Medicine and Faculty of Medicine, The University of Tokyo, Tokyo 113-0033, Japan; Department of Osteoimmunology, Graduate School of Medicine and Faculty of Medicine, The University of Tokyo, Tokyo 113-0033, Japan; Institute of Molecular Biotechnology of the Austrian Academy of Sciences (IMBA), Vienna, Austria; Department of Medical Genetics, Life Sciences Institute, The University of British Columbia, Vancouver, British Columbia, Canada; Department of Innovative Organoid Research, Helmholtz Centre for Infection Research, Braunschweig, Germany; Eric Kandel Institute, Department of Laboratory Medicine, Medical University Vienna, Vienna, Austria; Department of Immunology, Graduate School of Medicine and Faculty of Medicine, The University of Tokyo, Tokyo 113-0033, Japan; Division of Molecular Pathology, Research Institute for Biomedical Sciences, Tokyo University of Science, Chiba 278-0022, Japan; Department of Immunology, Graduate School of Medicine and Faculty of Medicine, The University of Tokyo, Tokyo 113-0033, Japan

**Keywords:** double-positive thymocytes, medullary thymic epithelial cells, thymus, transcriptome

## Abstract

The thymus is an organ required for T cell development and is also an eosinophil-rich organ; however, the nature and function of thymic eosinophils remain unclear. Here, we characterized the gene expression and differentiation mechanism of thymic eosinophils in mice. Thymic eosinophils showed a distinct gene expression profile compared with other organ-resident eosinophils. The number of thymic eosinophils was controlled by medullary thymic epithelial cells (mTECs). In Rag-deficient mice, the unique gene expression signature of thymic eosinophils was lost but restored by pre-T cell receptor signalling, which induces CD4^+^ CD8^+^ thymocyte differentiation, indicating that T cell differentiation beyond the CD4^−^ CD8^−^ stage is necessary and sufficient for the induction of thymic eosinophils. These results demonstrate that thymic eosinophils are quantitatively and qualitatively regulated by mTECs and developing thymocytes, respectively, suggesting that thymic eosinophils are a distinct, thymus-specific cell subset, induced by interactions with thymic cells.

## Teaser

Unique characteristics and interactions of thymic eosinophils

## Graphical Abstract

**Graphical Abstract:**
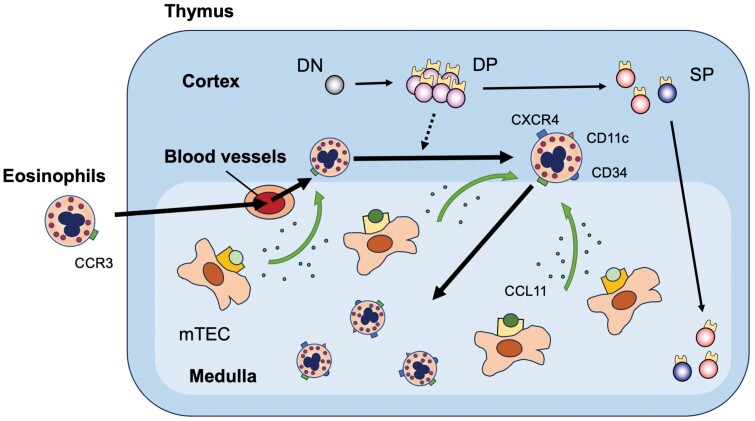

## Introduction

The thymus is a primary lymphoid organ where functional, yet self-tolerant T cells are generated through positive and negative selection (1). In the cortex of the thymus, CD4^−^ CD8^−^ (double negative, DN) thymocytes differentiate into CD4^+^ CD8^+^ (double positive, DP) thymocytes, which express the rearranged T cell receptor (TCR) (2). Upon the interaction with self-peptides and major histocompatibility complex (MHC) presented by cortical thymic epithelial cells (cTECs), DP thymocytes expressing functional TCRs differentiate into CD4^+^ CD8^−^ (CD4 single positive, CD4SP) or CD4^−^ CD8^+^ (CD8SP) thymocytes. This process is termed positive selection (3). The positively selected SP thymocytes migrate from the cortex to the medulla, where medullary TECs (mTECs) present diverse self-peptides so that autoreactive SP thymocytes are eliminated. This process is called negative selection (4).

The thymus is also an organ that hosts a number of eosinophils, a subpopulation of granulocytes. Eosinophils are primarily localized in peripheral organs including the spleen and lung, and are involved in T helper 2 (Th2) responses in parasite infection and allergy (5). Recently, in addition to effector eosinophils, the concept of homeostatic eosinophils has emerged, in which different types of eosinophils play a role in regulating immune responses and maintaining tissue homeostasis (6–8). For example, resident eosinophils in the small intestine are involved in the regulation of Th17 differentiation through the production of interleukin-1 receptor antagonist (IL-1Ra) to sustain homeostasis in the small intestine (9). Lung eosinophils are important in sensitization to antigens during the early stages of life, immune responses against viruses, and limitation of the Th2 response against house dust mites (10–12).

However, only a few studies have focused on thymic eosinophils. Previous studies have shown that thymic eosinophils are increased upon MHC-I-induced negative selection or irradiation-induced thymocyte depletion, but the precise mechanisms underlying this increase and physiological functions remain unclear (13, 14). One study demonstrated that the activation of thymic eosinophils correlates with Th2 cytokine expression in the human thymus (15), although this is not a thymus-specific event and also occurs in airway inflammation (16). Recent studies have focused on the unique roles of eosinophils in the thymus. Cosway *et al*. (17) reported that thymic eosinophils play a role in thymus regeneration after irradiation-induced damage. In human studies, CD34^+^ thymic eosinophils form immunological synapses with thymocytes (18), and thymocytes cocultured with thymic eosinophils increase the CD4/CD8 ratio of SP thymocytes (19). These studies suggest that thymic eosinophils have a unique, yet uncharacterized, role distinct from other tissue-resident eosinophils. However, how thymic eosinophils differ from other tissue-resident eosinophils at the molecular level and acquire their unique features remain unclear.

In this study, we evaluated the nature and differentiation mechanism of thymic eosinophils, focusing on the interaction with TECs and thymocytes. First, the number of thymic eosinophils correlated with the number of mTECs, suggesting the regulatory mechanism controlling the population size in the thymus of eosinophils by mTECs. Second, transcriptome analysis of eosinophils across tissues revealed that thymic eosinophils show a different gene expression signature than other tissue-resident eosinophils. Such a thymus-specific gene expression signature of eosinophils was associated with the differentiation of DP thymocytes in the thymus. These findings provide novel insights that thymic eosinophils may be a distinct population that crosstalk with thymus-specific cells, such as thymocytes and mTECs.

## Methods

### Mice

All mice were maintained under specific pathogen-free conditions, and all experiments were performed with the approval of the Institutional Animal Care and Use Committee of The University of Tokyo (Tokyo, Japan). Wild-type (WT) C57BL/6N mice were purchased from SLC Inc. (Hamamatsu, Shizuoka, Japan). *Foxn1*^*Cre*^ (20), *Tnfrsf11b*^*flox*^ (21), *Tnfrsf11a*^*flox*^ (22), *Cd40*^−*/*−^ (23), *Rag1*^−*/*−^ (24), *Rag2*^−*/*−^ (25), *Tcra*^−*/*−^ (26), and *Psmb11*^*G220R*^ (27) mice in the C57BL/6N background have been previously described. Age- and sex-matched littermates were used for all experiments. Mice were analysed at the age of 8–10 weeks in all experiments.

### Antibody treatment

Rag knockout (KO) mice (*Rag1*^−*/*−^ or *Rag2*^−*/*−^) were injected intraperitoneally with 200 μg hamster immunoglobulin G (IgG) (BioLegend, San Diego, CA, USA) or anti-mouse cluster of differentiation 3 epsilon (CD3ε) antibody (clone 145-2C11; BioLegend) in 300 μL sterile phosphate-buffered saline. After 10 days, mice were sacrificed and the thymus was collected.

### Histological analysis

Samples were embedded in optimal cutting temperature compound (Sakura Finetek Japan Co., Tokyo, Japan), frozen, and sliced into 5-μm-thick sections with a cryostat (Leica, Wetzlar, Germany). For immunohistochemical analysis, sections were air-dried, fixed in acetone, and stained with the following antibodies: anti-mouse CD11b (clone M1/70; BioLegend), anti-mouse sialic acid binding Ig-like lectin F (Siglec-F) (clone E50-2440; BD Biosciences, Franklin Lakes, NJ, USA), and anti-mouse keratin 14 (K14) (rabbit polyclonal; BioLegend). Images were obtained with the BZ-9000 fluorescent microscope (Keyence Co., Osaka, Japan) and analysed with BZ-H2A software (Keyence).

### Flow cytometry and cell sorting

Flow cytometry and cell sorting were performed with the FACSCanto II and FACSAria III systems (BD Biosciences). To prepare thymic stromal cells or thymic and lung eosinophils, tissues were digested with 0.01% Liberase TM (Roche, Basel, Switzerland) and 0.01% DNase I (Roche) (28). Thymocytes and splenocytes were prepared by mashing organs using glass slides. Before staining with fluorescent antibodies, cells were treated with an Fc blocker (anti-mouse CD16/CD32, clone 2.4G2; Tonbo Biosciences, San Diego, CA, USA). Cells were stained with a mixture of the antibodies at a final concentration of 0.5–10 μg/mL in the dark at 4°C. Antibodies used were Siglec-F/CD170-PE (clone E50-2440; BD Biosciences), CD11b-APC (clone M1/70; BioLegend), I-A/I-E-FITC (clone M5/114.15.2; BioLegend), CD11c-PE-Cy7 (clone N418; BioLegend), CD90.2(Thy1.2)-PacificBlue (clone 53-2.1; BioLegend), C-C motif chemokine receptor 3 (CCR3)/CD193-biotilynated (clone J073E5; BioLegend), streptavidin-APC-Cy7 (BioLegend), CD45-PacificBlue (clone 30-F11; BioLegend), epithelial cellular adhesion molecule (EpCAM)-PE-Cy7 (clone G8.8; BioLegend), Ly51-AlexaFluor647 (clone 6C3; BioLegend), Ulex europaeus agglutinin I (UEA-1)-biotinylated (Vector Laboratories Inc., Newark, CA, USA), CD4-APC (clone GK1.5; BioLegend), and CD8a-APC-Cy7 (clone 53-6.7; BioLegend). 7-aminoactinomycin D (7AAD) was used to exclude dead cells. Data were analysed using FlowJo software (Tree Star, Ashland, OR, USA). To isolate eosinophils, Siglec-F^+^ cells were enriched using a PE-conjugated antibody for Siglec-F (clone E50-2440; BD Biosciences) and a magnetic bead-conjugated antibody specific for PE (Miltenyi Biotec, Gaithersburg, MD, USA). Then CD11b^+^ Siglec-F^+^ cells were sorted using the FACSAria III cell sorter (>95% purity).

### RT-PCR

Total RNA was extracted from isolated cells using the RNeasy Plus Micro Kit (QIAGEN, Hilden, Germany) and reverse transcribed with SuperScript III (Invitrogen, Thermo Fisher Scientific, Waltham, MA, USA). Quantitative PCR (qPCR) was performed with THUNDERBIRD Next SYBR qPCR Mix (TOYOBO, Osaka, Japan) and the StepOnePlus Real-Time PCR System (Applied Biosystems, Thermo Fisher Scientific). The relative mRNA expression was normalized to *Gapdh* mRNA levels.

### Bulk RNA sequencing (RNA-seq) analysis

The cDNA libraries were prepared using the SMART-seq v.4 Ultra Low Input RNA Kit for Sequencing (Clontech Laboratories, Mountain View, CA, USA). Data were acquired on the Ion Proton Sequencer (Thermo Fisher Scientific, Waltham, MA, USA) and analysed using CLC genomics Workbench v.12 and R 4.3.0. Gene Ontology (GO) analysis was conducted using Metascape (29). The RNA-seq datasets generated in the present study are available in the Gene Expression Omnibus (GEO) database (www.ncbi.nlm.nih.gov/geo; Accession No. GSE266084). RNA-seq data of TECs (GSE65617) and bone marrow and small-intestinal eosinophils (GSE185070) were extracted from the GEO database.

### Single-cell RNA-seq analysis

Single-cell RNA-seq data of mTECs were extracted from the ArrayExpress dataset (Accession ID No. E-MTAB-8105). The reanalysis was performed using Seurat (v4.3.0) (30). Briefly, cells that contained a percentage of mitochondrial transcripts > 5% were filtered out. After log normalization, cell cycle scores were regressed out after assigning cell cycle scores via the CellCycleScoring function. Then 20 single-cell RNA-seq datasets were integrated with a combination of FindIntegrationAnchors and IntegrateData functions. The data were scaled and centred using these 2000 integration anchors, and principal component analysis was performed. The top 11 principal components were retained for shared nearest-neighbour graph construction (k = 20) and two-dimensional Uniform Manifold Approximation and Projection visualization based on jackstraw and elbow plots. Cell clustering was performed using the Louvain method (resolution = 0.4). The full source code for analysis is available in GitHub (heeps://github.com/nittatakeshi/).

### Statistical analysis

GraphPad Prism 9.4.1 and R 4.3.0 were used to perform the statistical analyses. The unpaired Student’s *t*-test was used for comparisons between two data sets. For comparisons of three or more groups, one-way analysis of variance was performed with Dunnett’s multiple comparison test or Tukey’s multiple comparison test. In all figures, bars represent the mean ± standard error of the mean (SEM).

## Results

### The number of thymic eosinophils is regulated by mTECs

**Figure 1.**
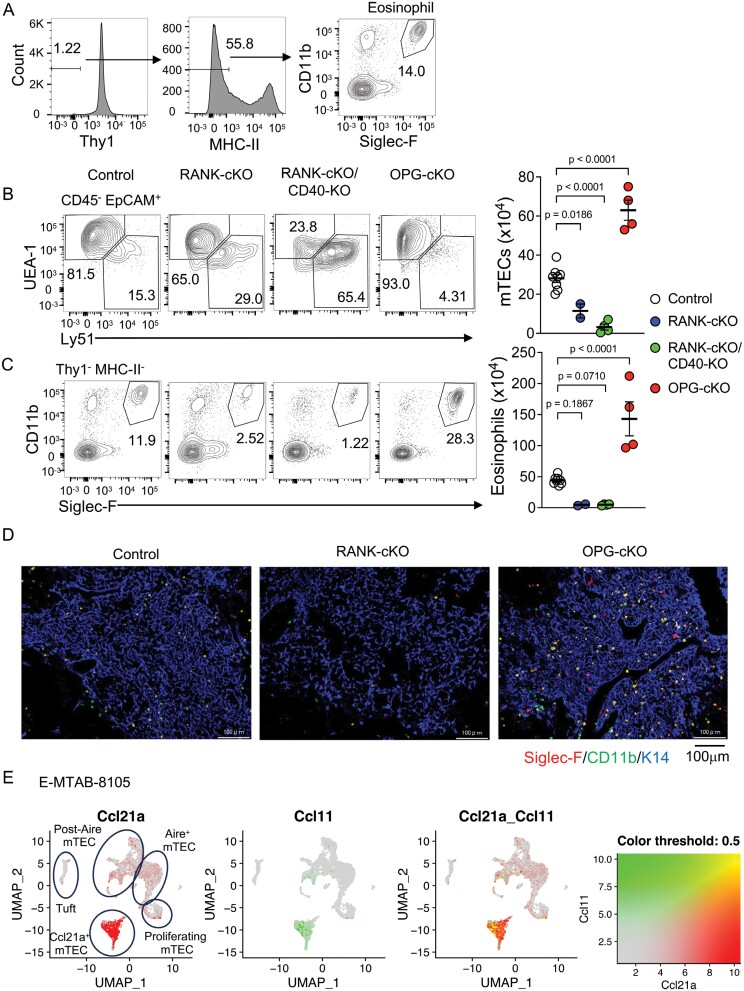
The number of thymic eosinophils is regulated by mTECs. (A) Gating strategy for eosinophils in the thymus by flow cytometry. Thy1^−^ MHC-II^−^ Siglec-F^+^ CD11b^+^ population was defined as eosinophils. (B and C) Flow cytometry analysis and quantification of TECs (B) and thymic eosinophils (C) from control (*Tnfrsf11a*^*fl/fl*^ or *Tnfrsf11b*^*fl/fl*^), RANK-cKO, RANK-cKO/CD40-KO, or OPG-cKO mice. (Left) Representative contour plots for Ly51 and UEA-1 of CD45^−^ EpCAM^+^ TECs (B) or for Siglec-F and CD11b of Thy1^−^ MHC-II^−^ population (C) are shown. (Right) In graphs, circles indicate individual mice and bars indicate the mean ± SEM. Control: *n* = 11, RANK-cKO: *n* = 2, RANK-cKO/CD40-KO: *n* = 4, OPG-cKO: *n* = 4. (D) Thymus sections from indicated mice were stained for Siglec-F, CD11b, and K14. The scale bars indicate 100 μm. (E) Feature plots in Uniform Manifold Approximation and Projection (UMAP) visualization were overlaid with the co-expression of *Ccl21a* and *Ccl11*. The single-cell RNA-seq data were extracted from E-MTAB-8105.

We performed quantitative analysis of thymic eosinophils in mice. Using flow cytometry, we defined Thy1^−^ MHC-II^−^ Siglec-F^+^ CD11b^+^ cells as thymic eosinophils (Fig. 1A) and estimated their number to be approximately 5 × 10^5^ per thymus in 8-week-old mice. This number was approximately the same as the dendritic cells and TECs.

Given that thymic eosinophils are localized in the medulla (31), we investigated the relationship between thymic eosinophils and mTECs, the major stromal cells of the medulla. The differentiation and proliferation of mTECs are induced by receptor activator of nuclear factor kappa B (RANK) and CD40 signalling, in response to RANK ligand (RANKL) and CD40 ligand (CD40L) expressed in SP thymocytes (32, 33). mTECs produce osteoprotegerin (OPG), a soluble decoy receptor for RANKL that inhibits RANKL–RANK signalling, conferring negative feedback regulation of mTEC development (21). Here, we examined the number of mTECs (CD45^−^ EpCAM^+^ Ly51^−^ UEA-1^+^) (Supplementary Figure 1A) and thymic eosinophils in *Foxn1*^*Cre/+*^*Tnfrsf11a*^*fl/fl*^ (RANK-cKO), *Foxn1*^*Cre/+*^*Tnfrsf11a*^*fl/fl*^*Cd40*^−*/*−^ (RANK-cKO/CD40-KO), and *Foxn1*^*Cre/+*^*Tnfrsf11b*^*fl/fl*^ (OPG-cKO) mice (20–23). Compared with control mice, the numbers of mTECs were reduced by 59% and 89% in RANK-cKO and RANK-cKO/CD40-KO mice, respectively, whereas mTECs were increased by 124% in OPG-cKO mice (Fig. 1B). The numbers of thymic eosinophils were reduced by 90% and 89% in RANK-cKO and RANK-cKO/CD40-KO mice, respectively (Fig. 1C). By contrast, thymic eosinophils were increased by 225% in OPG-cKO mice compared with control mice. The same results were observed in histological analyses (Fig. 1D). Almost no CD11b^+^ Siglec-F^+^ eosinophils were detected in the K14^+^ thymic medulla of RANK-cKO mice, whereas they were markedly increased in the medulla of OPG-cKO mice. These results indicate that the number of thymic eosinophils is positively correlated with the number of mTECs.

Eosinophils express the chemokine receptor CCR3, which is the receptor for the chemokine C-C motif chemokine 11 (CCL11)/eotaxin-1 (34). RNA-seq data (GSE65617) (35) revealed that the subset of mTECs expressing a low level of MHC-II (mTEC^lo^), highly expressed *Ccl11* (Supplementary Figure 1B). Single-cell RNA-seq reanalysis of total mTECs (E-MTAB-8105) (36) also showed that *Ccl11* is predominantly expressed in Ccl21a^+^ mTECs, the mTEC subset that produces chemokines attracting SP thymocytes into the medulla and is included in mTEC^lo^ (Fig. 1E, Supplementary Figure 1C). Together, these data support the belief that the number of thymic eosinophils is regulated by mTECs through the CCL11–CCR3 chemokine axis.

### Thymic eosinophils show specific gene expression compared with eosinophils from other organs

**Figure 2.**
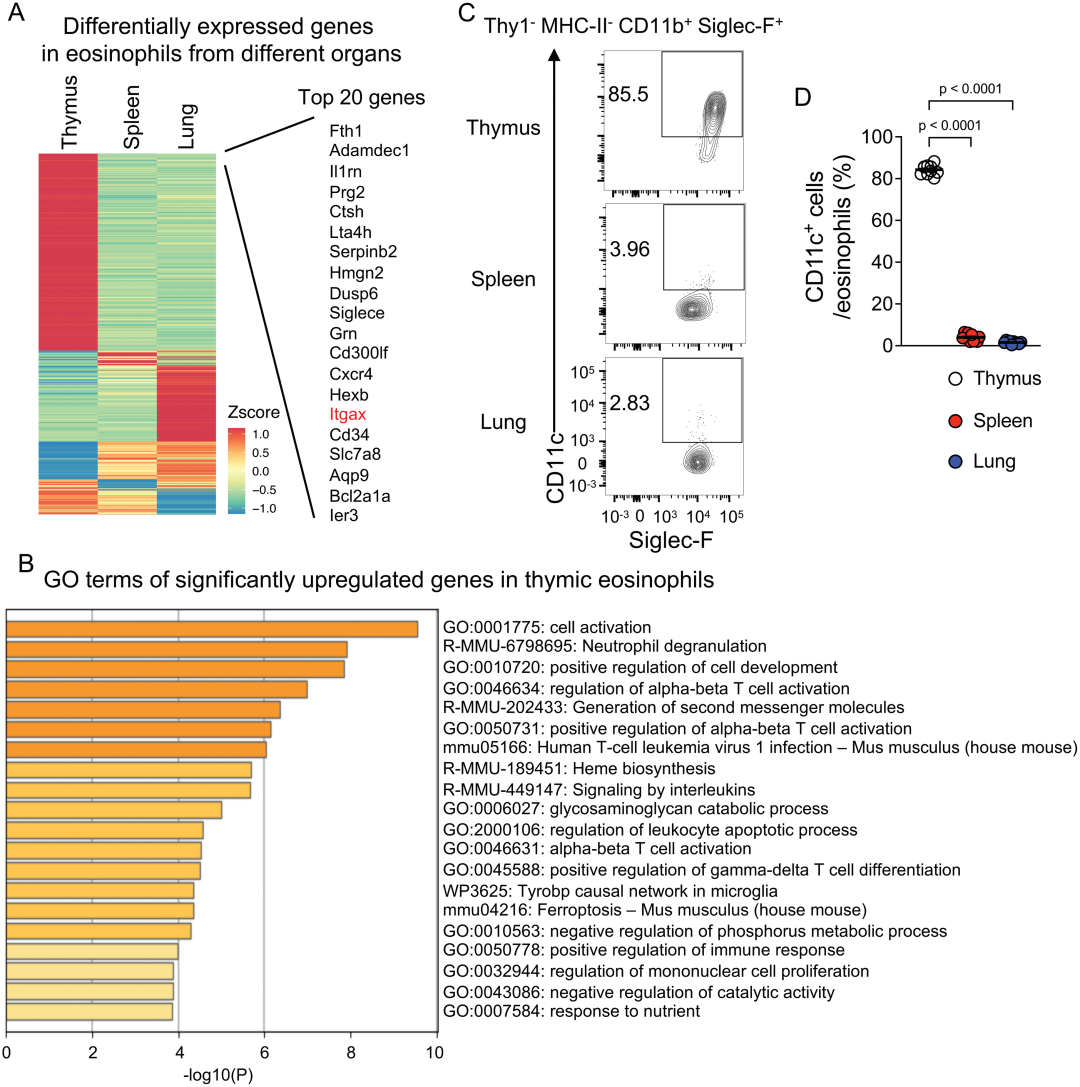
Thymic eosinophils show distinct gene expression compared with eosinophils from other organs. (A) Heatmap of differentially expressed genes (*P* < 0.05 using one-way analysis of variance with Dunnett’s multiple comparison test and fold change [FC] > 2 or < 0.5). Twenty genes with the highest expression are highlighted. The values are Z-scores of the means of reads per kilobase per million (RPKM) in the sorted eosinophils from each organ (*n* = 3 each). (B) GO analysis of genes significantly upregulated in thymic eosinophils compared with both spleen and lung eosinophils. (C) Flow cytometry analysis of eosinophils from indicated organs. Representative contour plots for Siglec-F and CD11c of gated eosinophils (Thy1^−^ MHC-II^−^ CD11b^+^ Siglec-F^+^) are shown. (D) The frequency of CD11c^+^ cells in the eosinophil population from the indicated organs (*n* = 10 each). Circles indicate individual mice and bars indicate the mean ± SEM.

To characterize thymic eosinophils at the gene expression level, we performed RNA-seq analysis on eosinophils sorted from the thymus, spleen, and lung (Supplementary Figure 2A). The expression trends of most eosinophil-specific genes, *Ccr3*, *Il5ra*, *Siglecf*, *Epx* (eosinophil peroxidase), and *Prg2* (proteoglycan 2) (37) and type 2 cytokines (*Il4, Il5, and Il13*), were shared among the eosinophils from different organs (Supplementary Figure 2B). However, the genes differentially expressed among different tissue eosinophils were also identified (Fig. 2A, Supplementary Figure 2C). The thymic eosinophil-specific genes included *Il1rn* (interleukin-1 receptor antagonist), *Cxcr4*, *Itgax* (integrin subunit alpha X, CD11c), *Cd34*, and *Bcl2a1d* (B cell lymphoma-2-related protein A1d). GO analysis revealed that thymic eosinophil-specific genes were associated with degranulation, regulation of αβT cell activation, and positive regulation of γδT cell differentiation (Fig. 2B). By contrast, spleen and lung eosinophils were associated with different GO terms (Supplementary Figure 2D and E). Spleen eosinophil-specific genes were limited. Lung eosinophils express genes associated with vascular formation. As the small intestine is also known to be an eosinophil-rich organ and a dataset of RNA-seq of small intestinal eosinophils and bone marrow eosinophils is available (38); GSE185070), we examined the expression of thymic eosinophil-specific genes in these two types of eosinophils (Supplementary Figure 2F). Small-intestinal eosinophils highly expressed several thymic eosinophil-specific genes including *Il1rn* and *Itgax*, but expressed another set of genes (e.g. *Fth1*, *Prg2*, *Hmgn2*, and *Gm*) to the same extent as bone marrow eosinophils, indicating that thymic and small-intestinal eosinophils are partially similar, but not identical, in gene expression. These data suggest that thymic eosinophils have specific characteristics and may be closely related to T cell selection and differentiation.

To detect the characteristics of thymic eosinophils, the expression of cell surface proteins was examined by flow cytometry. As the RNA-seq results indicated (Fig. 2A), the expression of CD11c (encoded by *Itgax*) was significantly higher in thymic eosinophils than in other tissue-resident eosinophils (Fig. 2C and D), consistent with previous study (13). Thereafter, this study used CD11c as a cell surface marker of thymic eosinophils.

### CD11c^+^ thymic eosinophils are not detected in Rag-KO mice

**Figure 3.**
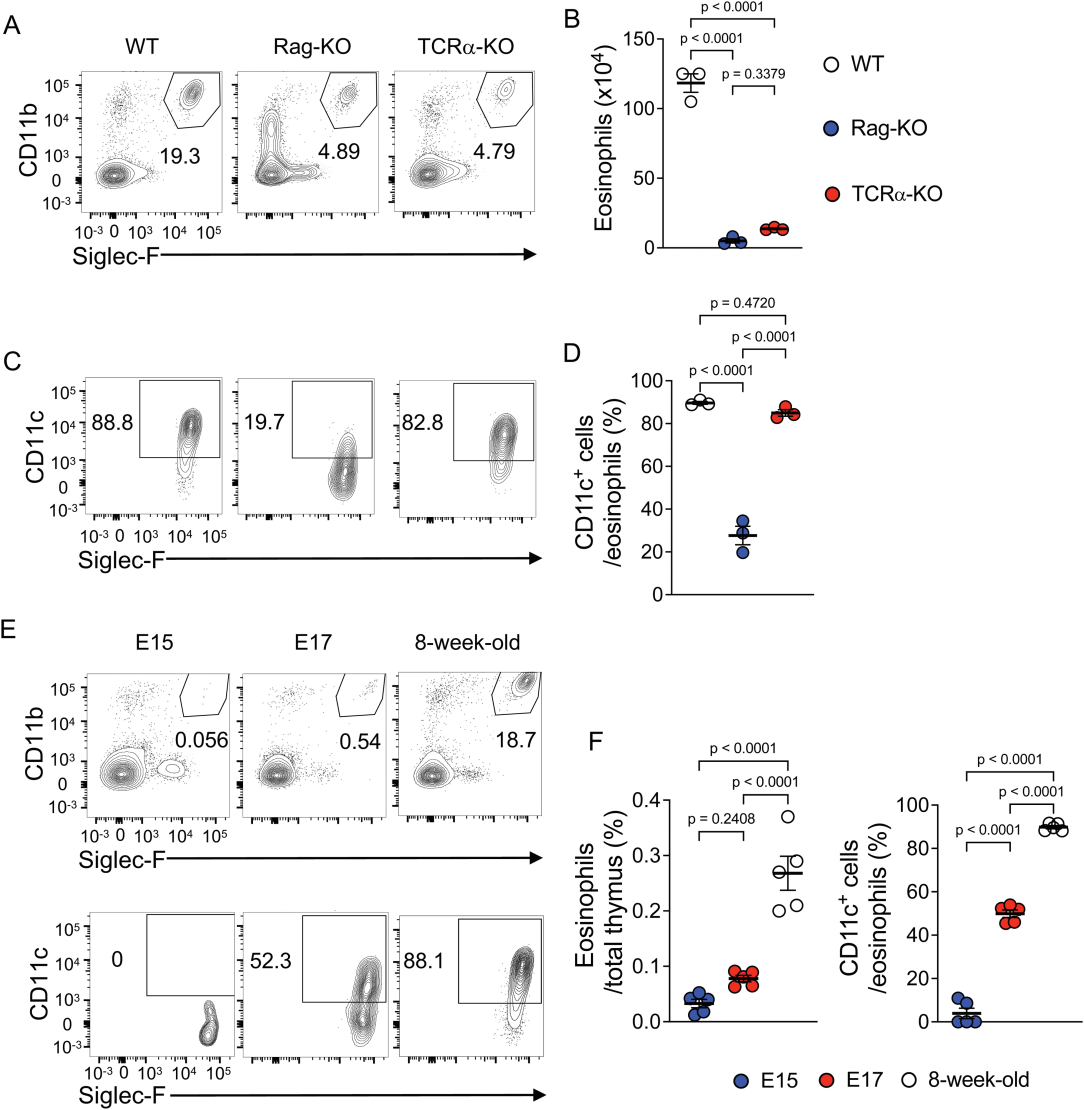
Thymic eosinophils lost CD11c expression in Rag-KO mice. (A and C) Flow cytometry analysis of eosinophils from WT, Rag-KO, and TCRα-KO mice. Representative contour plots for Siglec-F and CD11b of the Thy1^−^ MHC-II^−^ population (A) and for Siglec-F and CD11c of gated eosinophils (Thy1^−^ MHC-II^−^ CD11b^+^ Siglec-F^+^) (C) are shown. (B and D) The number of eosinophils (B) and the frequency of CD11c^+^ cells in the eosinophil population (D) from the indicated mice (*n* = 3 each). (E) Flow cytometry analysis of eosinophils from E15, E17, and 8-week-old mice. Representative contour plots for Siglec-F and CD11b of the Thy1^−^ MHC-II^−^ population (upper) and for Siglec-F and CD11c of gated eosinophils (Thy1^−^ MHC-II^−^ CD11b^+^ Siglec-F^+^) (lower) are shown. (F) The number of eosinophils (left) and the frequency of CD11c^+^ cells in the eosinophil population (right) from the indicated mice (*n* = 5 each). Circles indicate individual mice and bars indicate the mean ± SEM.

Our RNA-seq data suggested that thymic eosinophils may be associated with T cell selection and differentiation in the thymus (Fig. 2B). A recent study showed that the development of thymocytes is important for the heterogeneity of cTECs, while they are required for the development of thymocytes (39). To clarify whether such crosstalk exists between thymic eosinophils and the development of thymocytes, mutant mouse strains with abnormal T cell development, *Rag*^−*/*−^ or *Rag2*^−*/*−^ (Rag-KO) and *Tcra*^−*/*−^ (TCRα-KO) (24–26), were analysed. Thymocyte differentiation in Rag-KO mice is arrested in the DN stage, whereas that in TCRα-KO mice is arrested in the DP stage. Flow cytometry analysis revealed that the proportion of thymic eosinophils was significantly decreased in Rag-KO and TCRα-KO mice, likely reflecting the decreased mTECs in these mice (Fig. 3A and B, Supplementary Figure 3A). The proportion of CD11c^+^ eosinophils, however, was markedly decreased in Rag-KO mice but almost unaltered in TCRα-KO mice (Fig. 3C and D). These results indicate that DP thymocytes, which develop in TCRα-KO but not in Rag-KO mice, may have a crucial role in controlling thymic eosinophils.

These mice have developmental defects not only in conventional T lineage cells but also in other cell lineages. Rag-KO and TCRα-KO mice showed reduced development of TECs because of the loss of SP thymocytes expressing RANKL and CD40L. To determine whether mTECs influence the phenotype of thymic eosinophils, we evaluated mTEC-deficient mice (RANK-cKO/CD40-KO) and mice with increased mTECs (OPG-cKO). Neither RANK-cKO/CD40-KO nor OPG-cKO mice showed marked changes in the proportion of CD11c^+^ eosinophils (Supplementary Figure 3B). Thymic eosinophils sorted from these mice do not show significant changes in thymic eosinophil-specific gene expression (Supplementary Figure 3C). Furthermore, *Psmb11*^*G220R*^ (*TN*), a naturally occurring mutant strain that specifically lacks cTECs (27), showed a slight decrease in the proportion of CD11c^+^ eosinophils compared with WT mice (Supplementary Figure 3D), but not as prominent as Rag-KO mice. These results indicate that DP thymocytes rather than TECs are important for the regulation of thymus-specific gene expression in eosinophils.

During thymic ontogeny in WT mice, CD11b^+^ Siglec-F^+^ eosinophils were detected in the thymus at embryonic day 15 (E15) and their number increased thereafter. CD11c^+^ thymic eosinophils were barely detectable at E15 but were increased (about 50% of total thymic eosinophils) at E17 (Fig. 3E and F). This increase in CD11c^+^ thymic eosinophils coincided with the development of DP thymocytes from DN thymocytes, which occurs around E16 (40). This also supports the hypothesis that DP thymocytes promote the expression of CD11c in thymic eosinophils. Taken together, these data show that DP thymocytes play a pivotal role in the generation of thymic eosinophils.

### Enforced differentiation of DP thymocytes in Rag-KO mice induces thymic eosinophils

**Figure 4.**
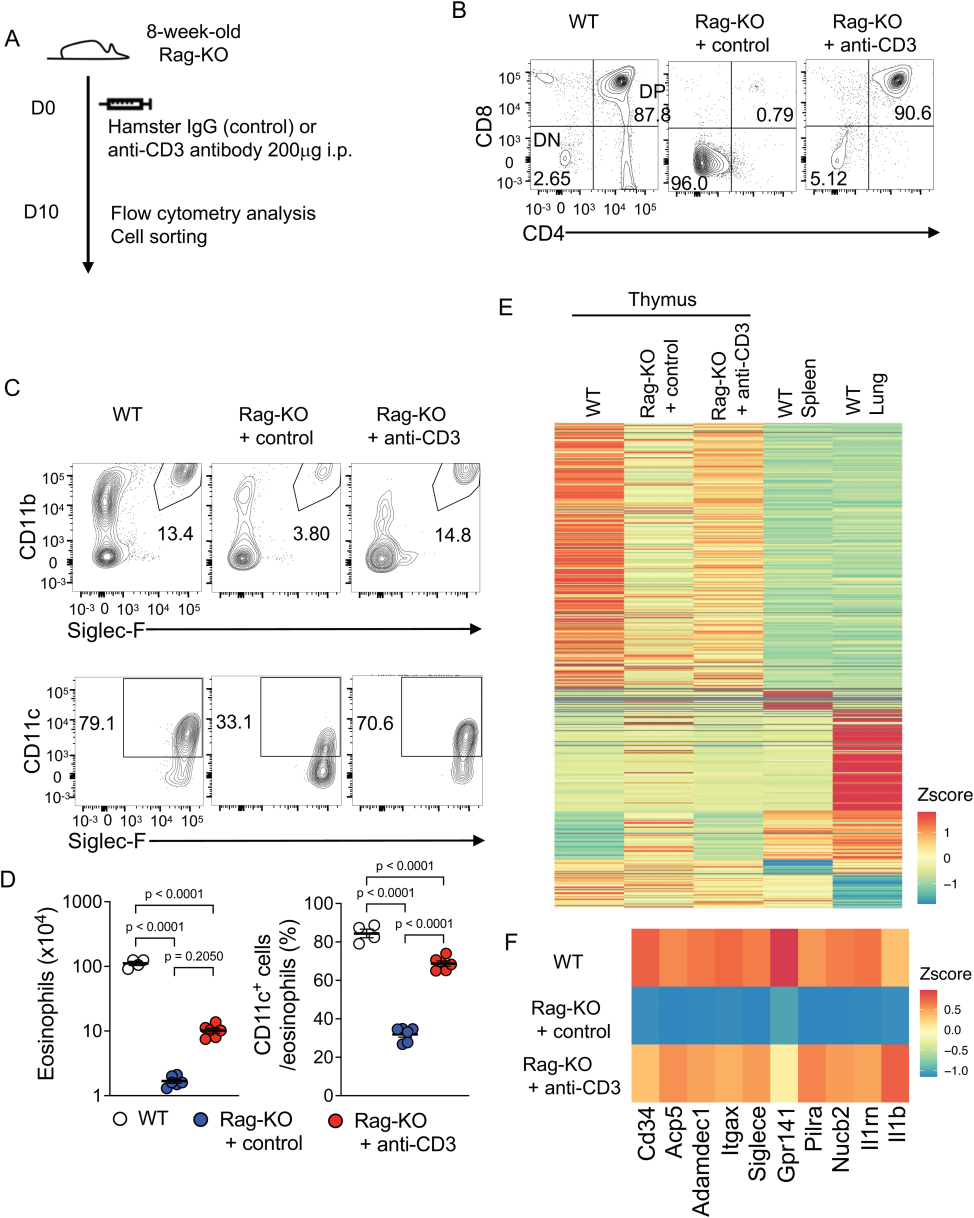
*In vivo* induction of DP thymocytes in Rag-KO mice rescued the thymic eosinophil-specific gene signature. (A) A schematic of the experiment. Eight-week-old Rag-KO mice were injected intraperitoneally (i.p.) with 200 μg hamster IgG (control) or anti-CD3 antibody. On day 10, mice were sacrificed. (B) Flow cytometry analysis of eosinophils from WT, Rag-KO mice injected with control (Rag-KO + control), and Rag-KO mice injected with anti-CD3 antibody (Rag-KO + anti-CD3). Representative contour plots for CD8 and CD4 of 7AAD^−^ live cells are shown. (C) Flow cytometry analysis of eosinophils from WT, Rag-KO + control, and Rag-KO + anti-CD3 mice. Representative contour plots for Siglec-F and CD11b of the Thy1^−^ MHC-II^−^ population (upper) and for Siglec-F and CD11c of gated eosinophils (Thy1^−^ MHC-II^−^ CD11b^+^ Siglec-F^+^) (lower) are shown. (D) The number of eosinophils (left) and the frequency of CD11c^+^ cells in the eosinophil population (right) from the indicated mice. Circles indicate individual mice and bars indicate the mean ± SEM. WT: *n* = 4, Rag-KO + control: *n* = 5, Rag-KO + anti-CD3: *n* = 5. (E and F) Heatmap of differentially expressed genes (the same as indicated in Fig. 2A) (E) and top 10 rescued genes by anti-CD3 treatment of Rag-KO mice (F). The values are Z-scores of the means of RPKM in each condition. WT: *n* = 3, Rag-KO + control: *n* = 2, Rag-KO + anti-CD3: *n* = 2.

To determine whether the differentiation of DP thymocytes can control thymic eosinophils, we utilized an *in vivo* experimental system, in which anti-CD3 antibody (clone: 2C11) was injected intraperitoneally into Rag-KO mice to stimulate pre-TCR signalling that induces differentiation towards DP thymocytes (Fig. 4A) (41). Ten days after the administration of anti-CD3 antibody, DP thymocytes were differentiated in Rag-KO mice (Fig. 4B). Under this condition, the proportion of CD11c^+^ cells in thymic eosinophils was significantly increased, while the control antibody had no effect (Fig. 4C and D). The number of eosinophils tended to increase but did not reach significance, and the number of mTECs did not change (Supplementary Figure 4), consistent with the findings that the number of thymic eosinophils is controlled by mTECs. To evaluate the effect on gene expression, RNA-seq analysis was performed on thymic eosinophils sorted from Rag-KO mice injected with control or anti-CD3 antibodies. The thymus-specific gene expression signature was lost in Rag-KO mice (Fig. 4E). Interestingly, most of the altered gene expression was restored in thymic eosinophils from anti-CD3-injected Rag-KO mice (Fig. 4E and F). These results indicate that the enforced differentiation of DP thymocytes induces the phenotype of thymic eosinophils *in vivo*.

## Discussion

In this study, we focused on the characteristics and developmental mechanisms of thymic eosinophils. Histological and flow cytometry analyses of mutant mice with altered TEC development showed that the number of thymic eosinophils was positively correlated with the number of mTECs. RNA-seq analysis revealed a unique gene expression signature in thymic eosinophils that differed from other tissue-resident eosinophils. Analysis of KO mice with defects in different stages of thymocyte development showed that DP thymocytes may play a pivotal role in the induction of a specific gene signature in thymic eosinophils.

Recent studies have paid attention to the concept of tissue-resident eosinophils, in which the nature and function of eosinophils differ by organ (6–8). However, such different subpopulations of eosinophils have been poorly characterized at the molecular level. This study revealed the gene expression profile of eosinophils across various tissues, supporting the idea that tissue-resident eosinophils have different characteristics. Compared with other tissue-resident eosinophils, thymic eosinophils highly express genes associated with γδT cell differentiation and αβT cell activation, suggesting their unique roles in the regulation of T lineage cells.

This study revealed the cell types that are essential for the regulation of thymic eosinophils. Our data suggest that mTECs are the major determinant of the number of thymic eosinophils, and this regulation may mediate the migration of eosinophils into the thymic medulla. RNA-seq data of a previous study (35) showed that mTEC^lo^ cells highly express *Ccl11*, also known as eotaxin-1, which is the main chemoattractant for eosinophils (42). A previous study also showed that no eosinophils are detectable in the thymus of mice lacking CCR3 (17), a chemokine receptor for CCL11, indicating that the CCL11–CCR3 axis is important for eosinophil distribution in the thymus. Our data suggest that mTEC^lo^ cells are the major source of CCL11 in the thymus and may regulate the number of thymic eosinophils. mTEC^lo^ cells are a heterogenous cell population containing a subset that produce chemokines, such as CCL21, which is essential for attracting SP thymocytes from the cortex to the medulla. Interestingly, single-cell RNA-seq analysis showed that the same population of mTECs expressing *Ccl21a* mainly produces *Ccl11* (36). These findings indicate that chemokine-producing mTECs simultaneously attract both SP thymocytes and eosinophils to the thymic medulla, suggesting a coordinated role for these two cell lineages in the thymic medulla. Additional studies are needed to reveal the molecular basis of mTEC-mediated eosinophil migration (i.e. to establish TEC-specific CCL11-deficient mice and elucidate where thymic eosinophils migrate from).

The present results also revealed the cellular link between acquisition of the unique phenotype of thymic eosinophils and T lineage cells. Unique markers and transcriptome signature in thymic eosinophils were lost in Rag-KO mice, in which T cell differentiation is arrested at the DN stage, but restored by enforced differentiation of DP thymocytes by pre-TCR signalling. These results clearly indicate that the differentiation of DP thymocytes is not only necessary but also sufficient for the induction of specific gene expression in thymic eosinophils. However, the detailed mechanism underlying how DP thymocytes influence thymic eosinophils has remained unclear. A recent study showed that the differentiation of DP thymocytes controls the heterogeneity of cTECs (39), suggesting that DP thymocytes indirectly promote thymic eosinophil induction via cTECs. Indeed, cTEC-deficient *TN* mice showed a slight but significant reduction of the proportion of CD11c^+^ thymic eosinophils. Thymic eosinophils are localized not only in the medulla but also in the cortico-medullary junction where cTECs and DP thymocytes reside. Therefore, future studies are needed to determine how cTECs and DP thymocytes affect eosinophils.

Regarding the role of thymic eosinophils, it has been reported that thymic eosinophils are recruited to the thymus upon MHC-I-restricted negative selection or irradiation-induced damage, suggesting that they are important in the clearance of apoptotic cells and regeneration of the thymus (13, 14, 17). The RNA-seq results in the current study indicated that eosinophils in all tissue express high levels of IL-4. This supports the idea that eosinophils contribute to the type 2 cytokine milieu in the thymus and other organs that control the regeneration of the damaged tissues. Our transcriptomic data also offer some insights into the functions of thymic eosinophils in the physiological state. One possible function is that thymic eosinophils may be a source of autoantigens for inducing the negative selection of autoreactive SP thymocytes. The thymus medulla contains different cell types, including fibroblasts (43), B cells (44), dendritic cells (45), as well as terminally differentiated mTECs (46), each of which produce cell type-restricted antigens to maximize the variety of self-antigens for T cell selection. Thymic eosinophils may be one of these cell types as a source of granulocyte-specific antigens, fulfilling the self-antigen repertoire in the medulla. Another possible function of thymic eosinophils is the regulation of thymocyte development. However, previous findings on thymic eosinophils and thymocytes have been inconsistent. ΔdblGATA mice deficient in eosinophil differentiation showed a significant reduction in CD8SP thymocytes, whereas IL-5-deficient mice, which also lack eosinophils, did not show such a phenotype (17). In contrast, *in vitro* coculture of human thymocytes and thymic eosinophils resulted in an increase in CD4SP thymocytes and a decrease in CD8SP thymocytes (19). Thus, the role of thymic eosinophils remains unclear. Further studies using mice specifically deficient in thymic eosinophils will be needed to elucidate the significance of the high abundance of unique eosinophils in the thymus.

In conclusion, this study showed that the number of thymic eosinophils was positively regulated by mTECs, while the specific gene expression was controlled by DP thymocytes (see the Graphical Abstract). Along with the transcriptomic data comparing various tissue-resident eosinophils, these findings suggest a new concept that this distinct population of eosinophils is closely related to the process of T cell development in the thymus. Thymic eosinophils are reportedly involved in dead cell clearance or thymus regeneration after damage, although the precise mechanisms remain unclear (14, 17). In addition to these pioneering findings on thymic eosinophils and well-known roles of eosinophils in parasite infection or in allergic response, our results suggest that thymic eosinophils may be a distinct non-T cell population that crosstalk with developing T lineage cells in the thymus. Based on the findings of this study, further studies will reveal the regulatory mechanisms and functions of thymic eosinophils, leading to a better understanding of T cell development and cellular basis of acquired immunity. This may provide novel insights into the development of therapeutic strategies to control infection, cancer, and autoimmune disorders.

## Supplementary data

Supplementary data are available at *International Immunology* Online.

dxae037_suppl_Supplementary_Figures_S1-S4
