# Supplementary material for: Synchronized development of thymic eosinophils and thymocytes
Source: Int Immunol. 2024 Jun 25;36(12):617–28. doi: 10.1093/intimm/dxae037 (PMC11562637; doi:10.1093/intimm/dxae037)
Supplement: dxae037_suppl_Supplementary_Figures_S1-S4 [file dxae037_suppl_supplementary_figures_s1-s4.docx]

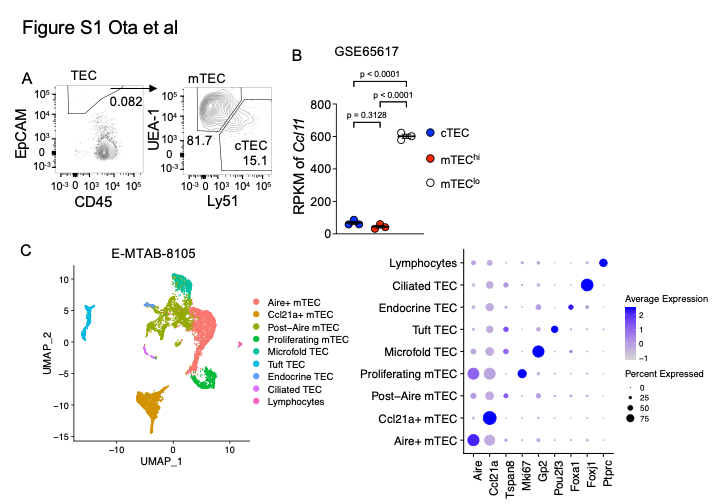
**Figure S1. An mTEC subset expresses *Ccl11*.**

(A) Gating strategy for TECs in flow cytometry. Gating on CD45^-^ EpCAM^+^ TECs, Ly51^-^ UEA-1^+^ and Ly51^+^ UEA-1^-^ populations were defined as mTECs and cTECs, respectively.

(B) The expression of *Ccl11* in each TEC subset. The data of WT mice were extracted from GSE665617. Circles indicate individual mouse and bars indicate the mean ± SEM (n = 3 each).

(C) (Left) TECs were clustered based on RNA expression in a UMAP plot. (Right) Dot plot showing the expression of marker genes used for annotation of each mTEC subset. The single-cell RNA-seq data were extracted from E-MTAB-8105.


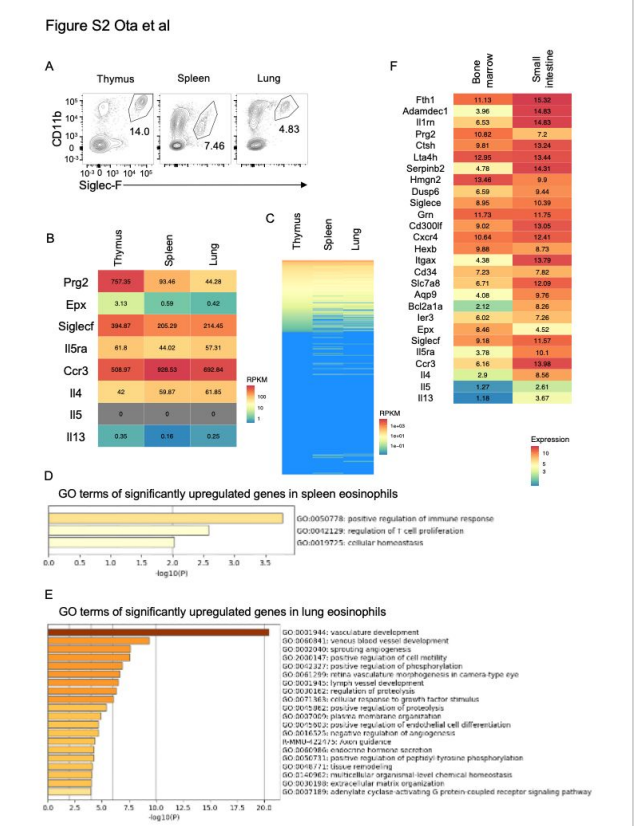


**Figure S2. Transcriptome of eosinophils in each organ.**

(A) Gating strategy for eosinophils from indicated organs in flow cytometry. Representative contour plots for Siglec-F and CD11b of Thy1^-^ MHC-II^-^ cell population are shown.

(B-C) Heatmap of eosinophil-specific genes (*Prg2, Epx, Siglecf, Il5ra, Ccr3*) (B) and all transcripts (C). The values are the means of RPKM in the sorted eosinophils from indicated organs.

(D-E) GO analysis of significantly upregulated genes (p < 0.05 using one-way analysis of variance with Dunnett’s multiple comparison test and FC > 2) in spleen (D) and lung (E) eosinophils compared with eosinophils in other organs.

(F) Heatmap of thymic eosinophil-specific genes (shown in Figure 2A) expressed in bone marrow eosinophils and small intestinal eosinophils (GSE185070). The values are the means of RPKM in eosinophils from each organ (n = 4 each).


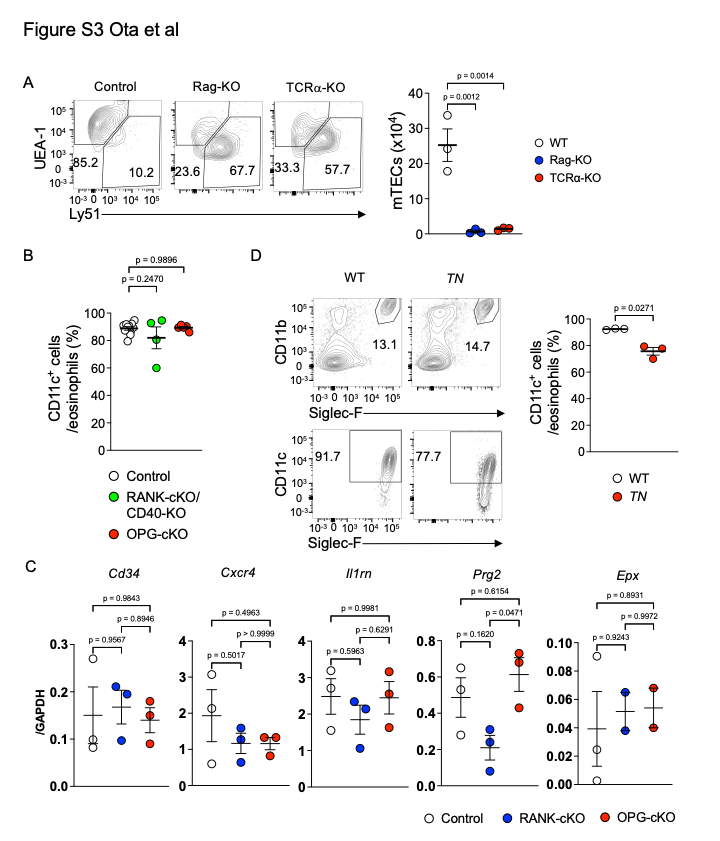


**Figure S3. Thymic eosinophils in TEC-deficient mice.**

(A) Flow cytometry analysis and the quantification of mTECs in WT, Rag-KO, and TCRα-KO mice. (Left) Representative contour plots for Ly51 and UEA-1 of TECs (CD45^-^ EpCAM^+^) are shown. (Right) The number of mTECs from indicated mice (n = 3 each).

(B) The frequency of CD11c^+^ cells in thymic eosinophil population from control (*Tnfrsf11a^fl/fl^* or *Tnfrsf11b^fl/fl^*) (n = 11), RANK-cKO/CD40-KO (n = 4), or OPG-cKO mice (n = 4).

(C) Quantitative RT-PCR analysis of thymic eosinophil-specific genes (*Cd34, Cxcr4, Il1rn, Prg2,* and *Epx*) in thymic eosinophils from control (*Tnfrsf11a^fl/fl^* or *Tnfrsf11b^fl/fl^*), RANK-cKO, and OPG-cKO mice (n=2-3 each). The relative mRNA expression was normalized to *Gapdh* mRNA levels.

(D) Flow cytometry analysis and quantification of thymic eosinophils from WT and *TN* mice. (Left) Representative contour plots for Siglec-F and CD11b of Thy1^-^ MHC-II^-^ population (upper) and for Siglec-F and CD11c of gated eosinophils (Thy1^-^ MHC-II^-^ CD11b^+^ Siglec-F^+^) (lower) are shown. (Right) The frequency of CD11c^+^ cells in the eosinophil population from indicated mice (n = 3 each).

Circles indicate individual mice and bars indicate the mean ± SEM.

**
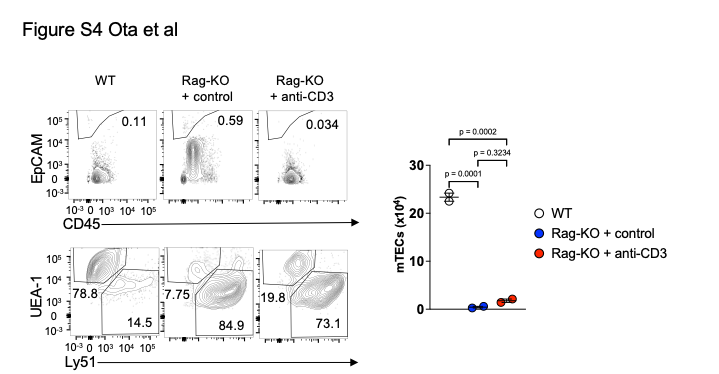
**

**Figure S4. Flow cytometry analysis of Rag-KO mice injected with anti-CD3.**

Flow cytometry analysis and the quantification of TECs from indicated conditions. (Left) Representative contour plots for CD45 and EpCAM of the 7AAD^-^ population (upper) and for Ly51 and UEA-1 of gated TECs (CD45^-^ EpCAM^+^) (lower) are shown. (Right) The number of mTECs from indicated condition (n = 2 each).
